# Immunogenicity and protective efficacy of inactivated coxsackievirus B4 viral particles

**DOI:** 10.1080/22221751.2024.2337665

**Published:** 2024-03-29

**Authors:** Tingfeng Wang, Chiyuan Wang, Lili Pang, Yujie Zhang, Shuxia Wang, Xiaozhen Liang, Zhong Huang

**Affiliations:** a Shanghai Institute of Immunity and Infection, Chinese Academy of Sciences, University of Chinese Academy of Sciences, Shanghai, People’s Republic of China; b Shanghai Institute of Infectious Disease and Biosecurity, Fudan University, Shanghai, People’s Republic of China

**Keywords:** Coxsackievirus B4, inactivated vaccine, E-particle, F-particle, neutralizing antibody, virus challenge

## Abstract

Coxsackievirus B4 (CVB4) is associated with a range of acute and chronic diseases such as hand, foot, and mouth disease, myocarditis, meningitis, pancreatitis, and type 1 diabetes, affecting millions of young children annually around the world. However, no vaccine is currently available for preventing CVB4 infection. Here, we report the development of inactivated viral particle vaccines for CVB4. Two types of inactivated CVB4 particles were prepared from CVB4-infected cell cultures as vaccine antigens, including F-particle (also called mature virion) consisting of VP1, VP3, VP2, and VP4 subunit proteins, and E-particle (also called empty capsid) which is made of VP1, VP3, and uncleaved VP0. Both the inactivated CVB4 F-particle and E-particle were able to potently elicit neutralizing antibodies in mice, despite slightly lower neutralizing antibody titres seen with the E-particle vaccine after the third immunization. Importantly, we demonstrated that passive transfer of either anti-F-particle or anti-E-particle sera could completely protect the recipient mice from lethal CVB4 challenge. Our study not only defines the immunogenicity and protective efficacy of inactivated CVB4 F-particle and E-particle but also reveals the central role of neutralizing antibodies in anti-CVB4 protective immunity, thus providing important information that may accelerate the development of inactivated CVB4 vaccines.

## Introduction

Coxsackievirus B4 (CVB4) is a member of the enterovirus genus within picornaviridae family [1]. The virus was first identified in 1979 [2], and since then it has been detected globally with endemic circulation [3–8]. CVB4 is transmitted through the fecal-oral route or the respiratory route. CVB4 infection is often mild or without symptoms, but sometimes it can result in a range of severe diseases or even death [9–12]. It has been documented that CVB4 infection can lead to severe neurological and cardiological complications such as meningitis, encephalitis, and myocarditis [13–19]. In addition, CVB4 has been reported to be closely associated with type 1 diabetes [20–28]. Moreover, CVB4 infection may cause severe respiratory diseases such as pneumonia [29–32]. Over the past ten years, CVB4 has become increasingly prevalent in China and is currently a major causative agent of hand, foot, and mouth disease (HFMD) in children [6, 12, 33, 34]. For example, a recent clinical survey shows that CVB4 is the second leading enterovirus serotype detected in the HFMD patient samples in Yunnan, China in 2013, 2015, and 2016, accounting for 16.4% of the severe HFMD cases [33]. The disease severity and increasing prevalence of CVB4 infection pose a serious threat to public health, underscoring the necessity to develop vaccines for CVB4.

Like other coxsackieviruses, CVB4 possesses a single-stranded positive-sense RNA genome of around 7400 nucleotides in length [35]. The viral genome encodes a single large polyprotein which can be processed into three precursor proteins, including one structural precursor (P1) and two non-structural precursors (P2 and P3). P1 can be further cleaved by a viral protease to produce capsid subunit proteins VP1, VP3, and VP0, among which VP0 may undergo autocleavage during virion maturation, yielding VP2 and VP4 [36]. A recent structural study shows that the capsid of CVB4 mature virion is made of 60 copies each of VP1, VP2, VP3, and VP4 subunit proteins [37].

Inactivated whole-virus vaccine has been demonstrated to be an effective strategy to develop vaccines against enteroviruses, as exemplified by the licensure of inactivated poliovirus vaccines and inactivated enterovirus 71 vaccines [38, 39]. For a given enterovirus, cell culture-produced viral particles naturally exist in two forms, infectious mature virion (also termed full particle or F-particle), which contains infectious viral RNA genome, and noninfectious procapsid (also termed empty particle or E-particle) without viral RNA [36]. The capsid of the enteroviral F-particle is made of VP1, VP2, VP3, and VP4 subunit proteins whereas that of the E-particle consists of VP1, VP3, and uncleaved VP0 [36]. These two particle forms may exhibit drastically different antigenicity, immunogenicity, and vaccine potency. Previous studies on poliovirus vaccine development have shown that the formalin-inactivated poliovirus F-particle displays the so-called “D antigen” and potently elicits protective levels of neutralizing antibodies whereas the E-particle predominantly expresses “C antigen” and is ineffective in eliciting neutralizing antibody response [40–44]. Consequently, only the F-particle or D antigen is considered to be the efficacious antigen in inactivated polio vaccines [45]. Similarly, Chong *et al.* reported that, for coxsackievirus A16 (CVA16), only the inactivated F-particle (named “R-particle” in the study), but not the E-particle (named “P-particle” in the study), was capable of inducing neutralizing antibodies in mice and rabbits [46]. Thus, the results from previous studies suggest that, in order to develop an effective inactivated whole-virus-based vaccine for a given enterovirus, it is crucial to characterize its F-particle and E-particle for immunogenicity and protective efficacy.

In the present study, we investigated the possibility of developing an inactivated whole-virus-based vaccine for CVB4. We prepared inactivated F-particle and E-particle of CVB4 as vaccine antigens and subsequently evaluated their immunogenicity and protective efficacy in a mouse model. Both CVB4 F-particle and E-particle were found to potently elicit neutralizing antibodies capable of protecting mice from lethal CVB4 infection. Higher titres of neutralizing antibodies were seen with the F-particle vaccine only after the third immunization.

## Materials and methods

### Cell and viruses

Human rhabdomyosarcoma cells (RD; ATCC #CCL-136) and Hela cells (ATCC #CCL-2) were cultured in Dulbecco’s Modified Eagle’s Medium (DMEM; Gibco, Thermo Fisher, USA) at 37°C in a 5% CO_2_ incubator, supplemented with 10% fetal bovine serum (FBS; Gibco). Coxsackievirus B4 prototype strain J.V.B (GenBank ID: X05690.1) was passaged in RD cells and titrated by the 50% tissue culture infectious dose (TCID_50_) assay using RD cells. CVB4 clinical strains GZ-R6 (GenBank ID: MZ540958.1) and GZ-HFM01 (GenBank ID: MZ540957.1) were isolated in a previous study [29]. These two CVB4 strains were propagated in Hela cells and the resulting virus stocks were titrated using Hela cells.

### Capsid subunit protein-specific polyclonal antibodies

Recombinant VP0, VP1, and VP3 proteins of CVB4/J.V.B. (GenBank ID: X05690.1) were individually expressed in *Escherichia coli*. Expression strategies and protocols were the same as those described for CVA16 in a previous study [47]. Purified recombinant proteins were quantified by Bradford assay. CVB4 VP0 protein was used to immunize mice for generating VP0-specific polyclonal antibodies. In the same way, the CVB4 VP1- and VP3-specific polyclonal antibodies were generated.

### Preparation of inactivated CVB4 F-particle and E-particle

RD cells grown to 90% confluency were rinsed twice with 10 mL of PBS buffer. The cells were inoculated with CVB4/J.V.B at multiplicity of infection (MOI) of 0.002 and then maintained in DMEM without FBS at 37°C until all the cells developed cytopathic effects (CPE). The culture was subjected to one freeze–thaw cycle and then clarified by centrifugation at 4500 rpm for 10 min at 4°C. Virus inactivation was carried out by treating virus-containing supernatants with a 1/2000 volume of β-propiolactone (Serva Electrophoresis, Heidelberg, Germany) for 24 h at 4°C, followed by incubation at 37°C for 2 h to permit β-propiolactone hydrolysis. Subsequently, the inactivated virus was layered onto a 20% sucrose cushion for partial purification and concentration. The resulting pellets were resuspended in PBS and then layered onto 10%−50% sucrose gradients for ultracentrifugation in a Beckman SW41Ti rotor at 39,000 rpm for 3 h at 4°C. After ultracentrifugation, 24 fractions were collected from top to bottom of the gradient and analyzed for the presence of CVB4 capsid subunit proteins by Western blot analysis using the VP0-specific polyclonal antibody. Based on the Western blotting results (in particular the VP0/VP2 banding pattern), the fractions containing F-particle or E-particle were separately pooled, followed by ultracentrifugation through a 20% sucrose cushion. The resulting viral particle pellets were resuspended in PBS and then stored at – 80°C until use. The final F-particle or E-particle antigen preparations were quantified by Bradford assay and analyzed by SDS-PAGE and western blotting with the indicated capsid subunit protein-specific polyclonal antibodies. The completeness of virus inactivation was tested by passage of the final antigen preparations on RD cells for periods of up to 2 weeks. No virus propagation was detected as indicated by the absence of CPE. Mock-infected RD cells were processed in the same way, yielding a negative control antigen for the immunization study.

### Electron microscopy

Purified CVB4 F-particle and E-particle samples were separately adsorbed on carbon-coated copper grids, negatively stained with 2% aqueous uranyl acetate, and then imaged with a Tecnai G2 Spirit transmission electron microscope (FEI, USA) at 120 kV.

### Mouse immunization

All mouse study protocols were approved by the Institutional Animal Care and Use Committee at the Shanghai Institute of Immunity and Infection, Chinese Academy of Sciences. Balb/c mice used for immunization were purchased from Vital River Laboratory Animal Technology (Beijing, China).

Before immunization, the antigens were mixed with aluminum hydroxide adjuvant (Alhydrogel®; Invivogen, USA; 500 μg / dose) to make the experimental vaccines. A single vaccine dose contained 5 μg antigen (CVB4 F-particle, CVB4 E-particle, or the control antigen) and 0.5 mg adjuvant in a final volume of 100 μl. Three groups (7 mice per group, 6-week-old) of female BALB/c mice were injected intraperitoneally (i.p.) with the experimental vaccines respectively, at weeks 0, 2, and 4. Blood samples from each mouse were collected at weeks 4 and 6. All mice were terminated at week 8 and blood was harvested. Sera were isolated from the blood and incubated at 56°C for 30 min to inactivate the complement.

### Antibody measurement

CVB4-specific antibodies in sera from immunized mice were measured by ELISA with *E.coli*-expressed CVB4 VP1 protein as the capture antigen. Briefly, 96-well ELISA plates were coated with the recombinant CVB4 VP1 proteins (50 ng/well) and incubated overnight. Then, the wells were blocked with 5% milk in PBST, followed by incubation with 50μl/well of serially diluted mouse sera at 37°C for 2 h and then with horseradish peroxidase (HRP)-conjugated anti-mouse IgG (Sigma, USA) at 37°C for 1 h. After colour development, the absorbance was measured at 450 nm. For each serum sample, its endpoint titre was defined as the highest serum dilution that had an absorbance >0.1 OD unit above the blank (the wells without addition of primary antibody).

### Neutralization assay

Serum samples were 2-fold serially diluted with DMEM containing 1% FBS. The CVB4/J.V.B virus stock was diluted to a working concentration of 2 TCID_50_ / μl. The neutralization assay was conducted using 96-well plates. In each well, 50 μl of diluted antiserum was mixed with 50 μl of the virus solution containing 100 TCID_50_ and incubated for 1 h at 37°C. Next, 50μl of cell suspension containing 20,000 RD cells was added to each of the wells containing the virus/antiserum mixtures and incubated at 37°C with 5% CO_2._ After 3 days, the cells were observed to evaluate the appearance of CPE. Neutralization titres were determined as the highest serum dilutions that could completely protect cells from CPE.

Cross-neutralization experiments were performed on Hela cells with the homologous strain CVB4/J.V.B. and two heterologous strains, CVB4/GZ-R6 or CVB4/GZ-HFM01. Briefly, 50 μl of 2-fold serially diluted serum was mixed with 50 μl of the virus (100 TCID_50_) and incubated at 37°C for 1 h. Next, 100 μl of Hela cell suspension was added to each well (15,000 cells/well) and incubated at 37°C. After 3 days, the cells were observed for CPE. The highest serum dilution showing complete inhibition of CPE was read as the neutralization titre.

### In vivo protection assays

Three groups of naive one-day-old ICR mice (n = 12-15) were i.p. injected with 30μl of pooled anti-F-particle, anti-E-particle, or control sera, respectively. One day later, all mice were inoculated i.p. with 3.15 × 10^6^ TCID_50_ of CVB4/J.V.B. The challenged mice were monitored daily for survival and clinical scores for 14 days. Clinical scores were graded as follows: 0, healthy; 1, lethargy or reduced mobility; 2, limb weakness; 3, limb paralysis; 4, death.

In another *in vivo* protection experiment, three groups of naive one-day-old ICR mice were pretreated with the pooled anti-F-particle, anti-E-particle, or control sera, and then challenged with CVB4/J.V.B. as described above. At 4 days post-infection, three surviving mice from each group were randomly selected and sacrificed. Limb muscles and brain tissues of the mice were taken, weighed and homogenized in DMEM with 1% FBS. The homogenate samples were clarified by high-speed centrifugation, and the resultant supernatants were used to determine viral loads by the TCID_50_ assay.

### Statistical analysis

All statistical analyses were performed using GraphPad Prism (V9) software. Survival curves were compared using Logrank test. Other results were analyzed using two-tailed *t*-test. Statistical significance was indicated as follows: ns, not significant (*P* ≥ 0.05); *, 0.01 ≤ *P* < 0.05; **, *P* < 0.01; and ***, *P* < 0.001.

## Results

### Preparation of inactivated CVB4 vaccine

**Figure 1.**
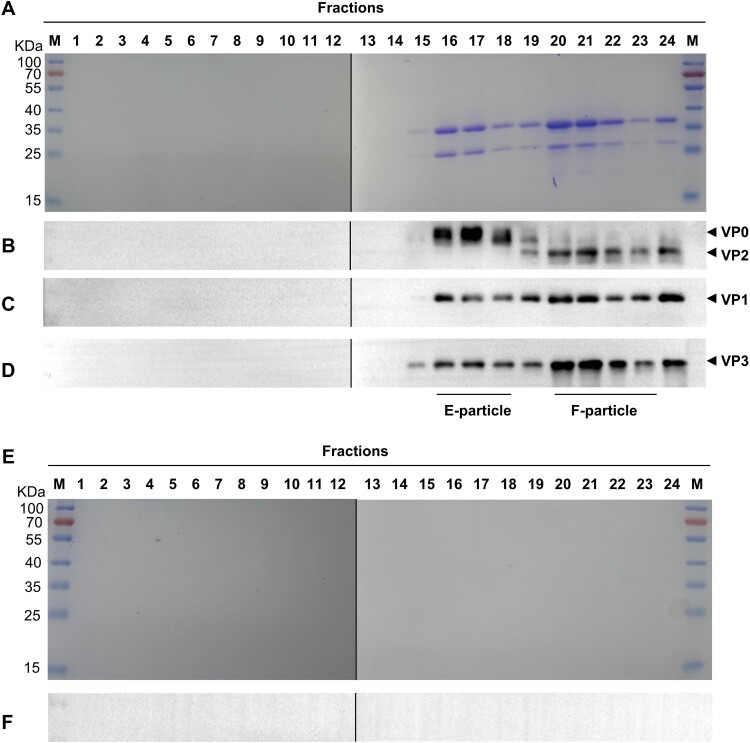
**Sucrose gradient analysis of inactivated CVB4 viral particles.** The lysates of mock – or CVB4-infected RD cells were inactivated and then sedimented on a 20% sucrose cushion. The resulting pellets were resuspended in PBS and then subjected to ultracentrifugation on 10%−50% sucrose gradient. Twenty-four gradient fractions were collected and then assayed. (**A-D**) SDS-PAGE and western blot analysis of the inactivated CVB4 samples. (**E-F**) SDS-PAGE and western blot analysis of the mock-infected control samples. Western blots were detected with anti-VP0 (**B and F**), anti-VP1 (**C**), or anti-VP3 (**D**) polyclonal antibodies, respectively.

The lysate of CVB4-infected RD cells was treated with β-propiolactone to inactivate the virus and then sedimented on a 20% sucrose cushion to enrich viral particles. The resulting pellets were resuspended in PBS and then subjected to ultracentrifugation on 10%−50% sucrose gradient. Twenty-four gradient fractions were collected and then analyzed for the presence and distribution of CVB4 capsid subunit proteins by SDS-PAGE and western blot assays (Figure 1A). The fast sedimenting fractions #16 to #24 produced protein bands that reacted with each of the anti-VP0, anti-VP1, and anti-VP3 polyclonal antibodies on western blots (Figure 1B-D). Notably, the anti-VP0 reacting bands in factions #16 to #18 were ∼40 kDa (expected molecular weight for uncleaved VP0) whereas those detected in fractions #20 to #23 were ∼35KDa (expected molecular weight for VP2) (Figure 1B). Based on the sedimentation profile and the VP0/VP2 banding pattern, we concluded that the fraction #16 to #18 and #20 to #23 contained E-particle and F-particle, respectively. Mock-infected RD cells were also identically processed and analyzed, however, the corresponding fractions #16 to #24 did not produce any visible protein band on SDS-PAGE or positive signal on western blots when detected with the CVB4 VP0-specific antibody (Figure 1E-F).

**Figure 2.**
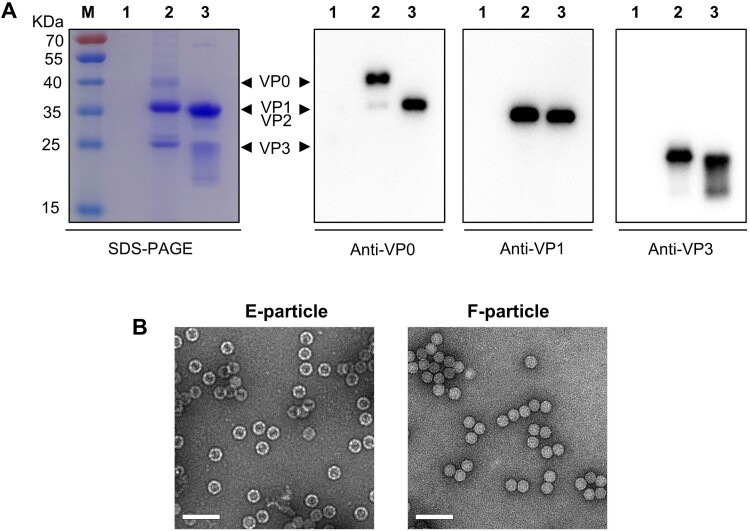
**Characterization of purified CVB4 E-particle and F-particle.** (**A**) SDS-PAGE and western blot analysis of purified antigens. Lane M, protein marker; lane 1, the control antigen prepared from uninfected cells; lane 2, the CVB4 E-particle preparation; lane 3, the CVB4 F-particle preparation. The detecting antibodies used in western blotting were indicated. (**B**) Negative stain electron microscopy of the CVB4 E-particle and F-particle preparations. Scale bar =100 nm.

The E-particle-containing fractions #16 to #18 and the F-particle-containing fractions #20 to #23 were separately pooled and then subjected to sedimentation through a 20% sucrose gradient, yielding the final E-particle and F-particle preparations, respectively. SDS-PAGE and Western blotting analysis showed that the E-particle was composed of VP0, VP1, and VP3 subunit proteins whereas the F-particle contained VP2, VP1, and VP3 proteins (Figure 2A). In contrast, no protein band was detected for the control antigen prepared from mock-infected RD cells (Figure 2A).

The E-particle and F-particle preparations were further analyzed by negative stain electron microscopy. Spheric particles with a diameter of around 30 nm were evident for both samples (Figure 2B). Notably, the particles of the E-particle preparation showed a heavily stained core, indicating they are indeed empty particles (Figure 2B). In contrast, the particles of the F-particle preparation did not have stained core (Figure 2B), in consistent with the nature of F-particle/mature virion. Collectively, the above data showed that inactivated CVB4 E-particle and F-particle antigens were successfully prepared.

### Immunogenicity comparison of inactivated F-particle and E-particle

**Figure 3.**
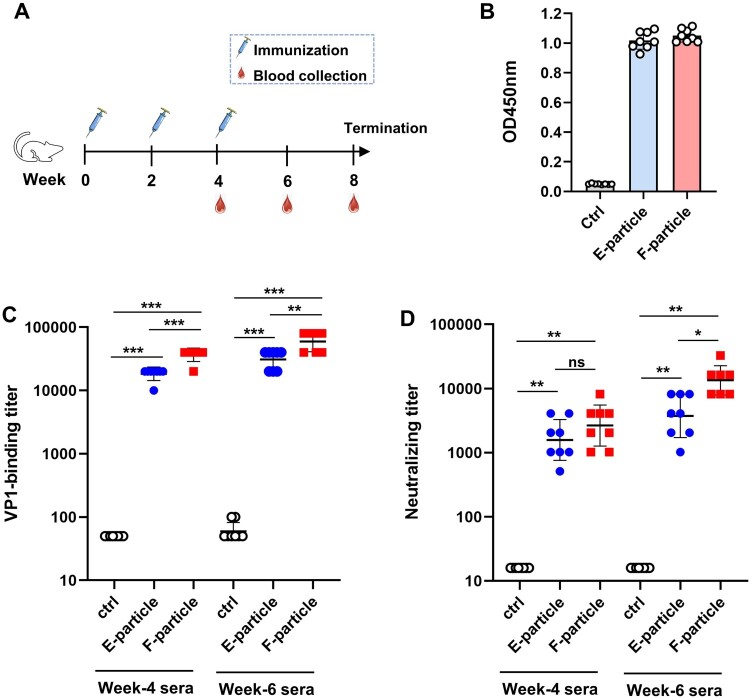
**Both E-particle and F-particle experimental vaccines potently elicited neutralizing antibodies in mice.** (**A**) Mouse immunization schedule. Three groups of BALB/C mice (n = 8) were injected i.p. with alum-formulated inactivated CVB4 E-particle, F particle, or the control antigen at weeks 0, 2, and 4. Serum samples were collected from individual mice at weeks 4 and 6. (**B**) The week-4 antisera were diluted 1:100 and analyzed for CVB4 VP1-binding activity by ELISA. Each symbol represents one mouse. (**C**) CVB4 VP1-binding titres of individual antisera collected at weeks 4 and 6. Each symbol represents one mouse. Sera that did not exhibit any binding activity at the lowest serum dilution (1:100) were assigned a titre of 50 for computation of geometric mean titre (GMT). GMTs ± SD for each group were shown. (**D**) Neutralization titres of the antisera against CVB4 strain JVB. Each symbol represents an individual mouse. Sera that did not exhibit neutralization at the lowest sera dilution (1:32) were assigned a titre of 16 for GMT computation. Neutralizing GMTs ± SD for each group were shown. *p* values were analyzed with *t*-test and indicated as follows: ns, no significant; *, *p *< 0.05; **, *p *< 0.01; ***, *p *< 0.001.

For immunogenicity studies, the inactivated CVB4 E-particle, F-particle, and the control antigen were separately formulated with the aluminum hydroxide adjuvant to make the experimental vaccines. Two groups of female Balb/c mice were injected i.p. with the E-particle (5μg/dose) and the F-particle (5μg/dose) vaccines, respectively, at weeks 0, 2, 4 (Figure 3A). Another group of mice was administered the control antigen in the same manner. Serum samples were collected at weeks 4 and 6 and analyzed for CVB4-specific antibodies by ELISA with the recombinant CVB4 VP1 protein as the capture antigen. As shown in Figure 3B, all mice immunized with the E-particle or the F-particle experimental vaccines developed significant CVB4 VP1-specific antibody responses after the second dose; in contrast, none of the mice injected with the control antigen had detectable CVB4 VP1-specific antibodies. At week 4, the F-particle-immunized mice showed higher VP1-binding antibody titres (GMT = 36680) compared to the E-particle-immunized ones (GMT = 18340), whereas all mice in the control group were sera-negative (Figure 3C). After the third immunization, the VP1-binding antibody titres of the two vaccine groups further increased with GMTs being 59440 and 30844 for the F-particle and E-particle groups, respectively, indicating an immune boosting effect of the vaccines (Figure 3C).

Individual sera were then analyzed for their neutralization potency against the live CVB4/J.V.B virus. The antisera from the control antigen-immunized mice did not show any neutralizing activity even at the lowest dilution tested (1:32) and was therefore assigned a titre of 16 for GMT computation; in contrast, all antisera in the E-particle or F-particle groups exhibited potent neutralization (Figure 3D). The neutralizing titres of the week-4 anti-E-particle sera ranged from 512 to 4096, comparable to those of the week-4 anti-F-particle sera (from 1024 to 8192) (Figure 3D). Elevated neutralizing antibody titres were detected in the week-6 antisera from the two vaccine groups with neutralizing GMTs being 3756 and 13440 for the E-particle and F-particle groups, respectively (Figure 3D).

To assess neutralization breadth, the week-6 antisera were pooled for each group and tested for neutralization of a panel of CVB4 strains, including the homologous strain J.V.B. (genotype I) and two heterologous strains, GZ-R6 (genotype V) and GZ-HFM01 (genotype V) [29]. The pooled control sera did not show neutralization activity against any of the three CVB4 strains at the lowest serum dilution tested (1:32); the pooled anti-E-particle sera effectively neutralized the homologous J.V.B. strain and the heterologous GZ-R6 and GZ-HFM01 strains with neutralizing titres being 4096, 4096, and 8192, respectively; whilst the pooled anti-F-particle exhibited nearly identical neutralization potency (neutralizing titre = 16,384) towards the three strains tested (Table 1). These data indicate that the serum antibodies induced by the inactivated CVB4/J.V.B. particles are broadly neutralizing towards diverse CVB4 genotypes/strains.

**Table 1.** Cross-neutralization capacities of the week-6 antisera.

Pooled week-6 antisera sample	Neutralization titres against CVB4 strains		
	J.V.B.	GZ-R6	GZ-HFM01
Control sera	<32	<32	<32
Anti-E-particle	4096	4096	8192
Anti-F-particle	16384	16384	16384

The lowest sera dilution test was 1:32.

All of the immunized mice were terminated at week 8 (4 weeks after the last immunization) and the antisera collected from individual mice were pooled for each group. The neutralizing titres against CVB4/J.V.B. were 4096 and 32768 for the week-8 anti-E-particle and anti-F-particle sera pools, respectively, whereas the week-8 control sera pool did not show neutralization at the 1:32 dilution. These pooled antisera were subsequently used for *in vivo* protection experiments.

### Protective efficacy of the experimental vaccines

**Figure 4.**
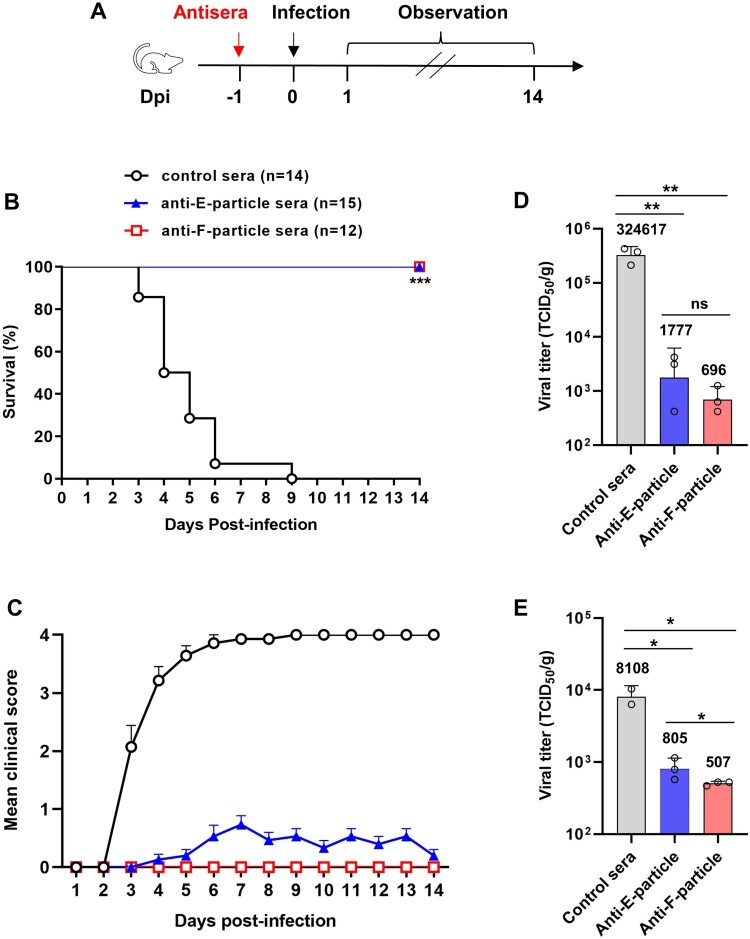
**Passive transfer of anti-E-particle or anti-F-particle sera effectively protected mice from lethal CVB4 challenge.** (**A**) Schedule of antisera transfer and virus challenge. groups of naive ICR mice (1-day-old) were injected i.p. with 30μl of the pooled anti-E-particle, anti-F-particle, or the control sera, respectively. One day later, the mice were inoculated i.p. with live CVB4 (3.15 × 10^6^ TCID_50_). Then, the mice were monitored daily for survival and clinical signs for a period of 14 days. (**B**) Survival rates of the mouse groups. The numbers of mice in each group were shown in brackets. Survival curves were compared using Logrank test and statistical significance was indicated as follows: ns, no significant; *, *p *< 0.05; **, *p *< 0.01; ***, *p *< 0.001. (**C**) Clinical scores of the mouse groups. Clinical scores were graded as follows: 0, healthy; 1, lethargy and reduced mobility; 2, limb weakness; 3, limb paralysis; 4, death. (**D-E**) Virus loads in the limb muscle (**D**) and brain (**E**) of the challenged mice at 4 days post-infection. Three mice from each treatment groups were randomly selected and their limb muscle and brain were collected and examined for CVB4 virus loads by the TCID_50_ assay. Each symbol represents an individual mouse. Geometric mean virus titres for each group were shown. Statistical analysis was performed using the *t*-test. *, *p *< 0.05; **, *p *< 0.01; ***, *p *< 0.001.

We adopted a passive immunization-virus challenge approach to evaluate the *in vivo* protective efficacy of the experimental vaccines. Groups of one-day-old ICR mice were passively immunized by i.p. injection of 30μl of the indicated antisera, one day later inoculated with a lethal dose of CVB4/J.V.B, and subsequently observed for 14 days (Figure 4A). The antisera used for passive transfer were the pooled antisera collected at week 8 in the active immunization experiment (Figure 3) and the neutralizing titres of the pooled anti-E-particle and anti-F-particle sera were 4096 and 32768, respectively. As shown in Figure 4B-C, the mice that had been given the control sera began to show signs of illness at 2 days post-infection (dpi), and all of them died by 9 dpi; in contrast, all of the mice treated with either the anti-F-particle or the anti-E-particle sera survived with no disease or only mild symptom observed in a proportion (10/15) of mice in the anti-E-particle group. The above data demonstrate that neutralizing antibodies elicited by either inactivated F-particle or E-particle vaccines can protect mice against lethal CVB4 infection.

To investigate how the antisera conferred protection *in vivo*, we performed another antisera transfer/virus challenge experiment and then examined virus loads in mouse tissues/organs at 4 dpi. Because the infected mice in the control group showed limb paralysis prior to death (Figure 4C), we thus chose limb muscle and brain as the target tissue/organ for measurement of virus loads. As shown in Figure 4D, the amount of replicating CVB4 virus (geometric mean titre = 324,617 TCID_50_ per gram tissue) in the limb muscle of the control sera-pretreated, CVB4-inoculated mice was 183- and 466-fold higher than those detected in the mice pretreated with the anti-E-particle or the anti-F-particle sera, respectively. Similarly, the virus load in the brain of the mice in the anti-E-particle or the anti-F-particle groups decreased by 10 and 16 folds, respectively, as compared to that of the control sera-pretreated mice (geometric mean titre = 8108 TCID_50_ per gram tissue) (Figure 4E). Taken together, the above data demonstrate that the passively transferred anti-E-particle and anti-F-particle sera can potently inhibit CVB4 infection *in vivo*, thereby protecting mice from infection-induced severe disease and death.

## Discussion

Accumulating clinical evidence has shown that CVB4 infection is associated with a broad range of diseases with severe consequences, underscoring an unmet need for effective CVB4 vaccine [48]. In this study, we demonstrated for the first time that both inactivated CVB4 F-particle and E-particle are strong vaccine candidates with proven immunogenicity and protective efficacy in preclinical trials. Before our work, there were only several published studies concerning CVB4 vaccine development. An early work showed that immunization with formalin-inactivated CVB4 elicited humoral and cellular immune responses towards the immunizing virus antigen in non-obese diabetic (NOD) mice and delayed onset of diabetes, however, it did not investigate the protective efficacy of the vaccine against CVB4 infection [49]. Recently, Stone *et al.* reported that formalin-inactivated CVB4 alone or in a hexavalent formulation containing inactivated CVB serotypes 1–6 could induce CVB4-neutralizing antibodies and protect against acute CVB4 infection in NOD mice [50]. In the present study, we evaluated the protective efficacy of inactivated CVB4 vaccine candidates by using a lethal CVB4 infection mouse model, which is more stringent than the NOD mouse model used previously. We found that β-propiolactone-inactivated CVB4 experimental vaccines elicited high-titre neutralizing antibodies and passive transfer of 30μl of the anti-CVB4 sera was sufficient to protect recipient mice from lethal CVB4 infection (Figure 4). Our data validate that inactivate whole-virus vaccine is a viable approach to developing effective CVB4 vaccines. The findings from the present and above-mentioned studies should have positive impact on the development of inactivated CVB4 vaccines.

It is well known that cell culture-derived enteroviruses usually present two particle forms, F-particle (or mature virion) and E-particle (or empty capsid) [36]. For a given enterovirus, its F-particle and E-particle could be drastically different in immunogenicity and vaccine potency. For instance, the poliovirus F-particle exhibited protective immunogenicity whereas the E-particle was considered non-immunogenic [40, 41]. Similarly, for CVA16, only the F-particle, but not the E-particle, was found to be capable of inducing CVA16-specific neutralizing antibody responses in both mouse and rabbit immunogenicity studies [46]. In sharp contrast, in the present study, we found that immunization of mice with either inactivated CVB4 F-particle or E-particle induced neutralizing antibodies at levels way above those required for complete protection against lethal virus challenge, despite relatively lower neutralizing antibody titres were seen with the E-particle vaccine after the third immunization (Figure 3D). It remains unknown why the E-particles from different enteroviruses had so varied vaccine potency. Nonetheless, our data indicate that both CVB4 F-particle and E-particle can be used as the source of inactivated CVB4 vaccine.

In our *in vivo* protection tests, all of the mice that had been passively immunized with 30μl of the anti-E-particle or anti-F-particle pooled sera survived from lethal CVB4 infection. However, the anti-F-particle sera recipient mice showed less clinical score and lower virus load in the brain compared to the mice pretreated with the anti-E-particle (Figure 4C-E), indicating that the anti-F-particle sera are more efficacious *in vivo* than the anti-E-particle sera. The levels of *in vivo* protection provided by the two input antisera appear to correlate with their *in vitro* neutralization titres (4096 and 32768 for the anti-E-particle and anti-F-particle pooled sera, respectively). Collectively, these data indicate that neutralizing antibody is a major, if not the sole, protective mechanism for CVB4 vaccines.

Previous studies have shown that inactivated CVB4 vaccine candidates had good safety profiles in animal models [49, 50]. In particular, Stone *et al.* reported that the hexavalent inactivated vaccine covering CVB1-6 had no adverse effects on either weight or blood glucose levels of vaccinated NOD mice and rhesus macaques when compared to control animals [50]. In the present study, robust protective efficacies of inactivated CVB4 experimental vaccines were clearly demonstrated using a lethal virus challenge mouse model. Together, these results warrant further development of inactivated CVB4 vaccines. Further work must be done in order to advance an inactivated CVB4 vaccine candidate to the next stage of development. First, a regulation agency-approved cell substrate that also permits efficient CVB4 propagation needs to be identified, as RD cells used in the present proof-of-concept study are tumorigenic cell lines and therefore are unsuitable for vaccine production. Second, more detailed immunogenicity analyses (such as dose–response relationship, time-effect relationship, and immune persistence) of inactivated CVB4 vaccine candidates need to be carried out in mice and perhaps in nonhuman primate models, so as to identify an optimal immunization scheme.

Besides inactivated whole-virus vaccines, recombinant virus-like particles (VLPs) are a promising alternative strategy to develop vaccines against enteroviruses [51]. Co-expression of P1 and 3CD of a given enterovirus in a recombinant system could lead to the formation of VLPs consisting of correctly processed VP0, VP1, and VP3 subunit proteins [52–55]. As the CVB4 E-particle is also made of VP0, VP1, and VP3 and has now been shown to elicit a protective immune response in the present study, we speculate that, if made, recombinant CVB4 VLP resembling the E-particle in morphology and protein composition will also be immunogenic and protective. Surprisingly, it was recently reported that recombinant CVB4 VP1 protein alone could self-assemble into VLPs with a diameter of around 25 nm, and this VP1-VLP vaccine could induce neutralizing antibodies and protect mice from lethal virus challenge [56]. In the future, this VP1-VLP should be compared in parallel with the VP0/VP1/VP3-coassembled VLP for their immunogenicity and protective efficacy, to identify an optimal CVB4 VLP vaccine candidate for further development.

In summary, the present study defines the immunogenicity and protective efficacy of inactivated CVB4 F-particle and E-particle. It reveals the central role of neutralizing antibodies in anti-CVB4 protective immunity, therefore providing important information for the development of inactivated whole-virus vaccines and recombinant VLP vaccines against CVB4.

## Data Availability

All data generated or analyzed during this study are included in this published article.
